# The complete chloroplast genome of *Horsfieldia hainanensis*, an Endangered species with extremely small populations

**DOI:** 10.1080/23802359.2019.1644556

**Published:** 2019-07-22

**Authors:** Yong Yang, Qiang Liu, Yu-Kai Chen, Yong Wang, Qing Chen

**Affiliations:** a Ministry of Education Key Laboratory for Ecology of Tropical Islands, College of Life Sciences, Hainan Normal University, Haikou, China;; b Bawangling National Nature Reserve, Changjiang, Hainan Province, China

**Keywords:** *Horsfieldia hainanensis*, Endangered plant, chloroplast genome, phylogenetic analysis

## Abstract

*Horsfieldia hainanensis* is an Endangered species with extremely small populations, inhabiting the dense forest in ravines. It has high development and utilization value in the seed oil, fatty acids, volatile oils, and liposoluble constituent. In this study, the complete chloroplast genome of *Horsfieldia hainanensis* was sequenced. It is 155,774 bp in size with a typical quadripartite structure: a large single-copy (LSC) region of 87,124 bp, a small single-copy (SSC) region of 20,612 bp, and 2 inverted repeat (IR) regions of 24,020 bp. The genome contains 117 genes, including 75 protein-coding genes, 34 tRNA genes, and 8 rRNA genes, 13 genes have one intron and 3 three genes have two introns. The overall GC contents of the chloroplast genome was 39.1%. The phylogenetic tree based 11 complete chloroplast genome sequences of Magnoliales shows that *H. hainanensis* is closely related to *Horsfieldia pandurifolia*.

*Horsfieldia hainanensis* Merrill (Myristacaceae) is an evergreen tree, which is mainly distributed in Guangxi, Hainan, Yunnan of China. It is an Endangered species with an extremely small population, inhabiting the dense forest in ravines at an altitude of 800–1200 meters. The population has been drastically reduced due to long-term utilization and unreasonable logging (Chen et al. 2014). The seed of *H. hainanensis* has a high oil content and the main fatty acids are Myristic acid and Lauric acid (Xu et al. 2012), which has high development and utilization value. In recent years, research on *H. hainanensis* has focused on the chemical constituents of volatile oils (Dang et al. 2009), liposoluble constituent (Liu et al. 2010), community structure and characteristics (Jiang et al. 2016), growth and physiological characteristics (Fu et al. 2018).

Using Illumina sequencing technology, the complete chloroplast genome of *H. hainanensis* was sequenced and its characteristic was analyzed. It can provide valuable data for phylogenetic inference or the population history of *Horsfieldia*, which can also aid in the utilization of the genetic resources of *Horsfieldia* as a high utilization value tree.

Fresh leaves of *H. hainanensis* were collected in Bawangling National Nature Reserve, Hainan Island (N19°04′24, E109°09′05) in China. The voucher specimens of *H. hainanensis* were deposited at the botany laboratory of Hainan normal University with the accession number Yang Y-201903, Haikou, China. Total genome DNA was extracted from fresh leaves by the modified CTAB method (Doyle 1987) and then a library with insertion size of ∼500 bp was constructed. About 5.0 Gb of sequence date was generated from high-throughput DNA sequencing on Illumina Hiseq2500 platform. Using a partial *rbcL* gene sequence of *Horsfieldia pandurifolia* (Genbank accession NC042225) as seed, the chloroplast genome was assembled using the program SOAPdenovo (Luo et al. 2012). The assembled chloroplast genome sequence was then annotated using DOGMA program (Wyman et al. 2004).

The complete chloroplast genome sequence of *H. hainanensis* (GenBank accession MK948430) was 155,774 bp in size with a typical quadripartite structure: a large single-copy (LSC) region of 87,124 bp, a small single-copy (SSC) region of 20,612 bp, and two inverted repeat (IR) regions of 24,020 bp. The overall GC contents of the chloroplast genome were 39.1%. In total, 117 unique genes were annotated, including 75 protein-coding genes, 8 rRNA genes, and 34 tRNA genes. In those genes, 13 genes contains one intron (*atpF*, *ndhB*, *petB*, *petD*, *rpl2*, *rpoC1*, *rps16*, *trnA-UGC*, *trnG-GCC*, *trnI-GAU*, *trnK-UUU*, *trnL-UAA*, and *trnV-UAC*) and 3 genes contains two introns (*clpP*, *rps12,* and *ycf3*).

To understand the phylogenetic position of *H. hainanensis* in the order Magnoliales, we aligned 11 complete chloroplast genome sequence download from NCBI by MAFFT (Katoh and Standley 2013) and then constructed a neighbor-joining tree using MEGA7 (Kumar et al. 2016) with 1000 bootstrap replicates and set *Drimys granadensis* as an outgroup. The result in Figure 1 shows that *H. hainanensis* was most close with *Horsfieldia pandurifolia*. Our chloroplast genome data of *H. hainanensis* will provide essential and useful information for an evolutionary study about this Endangered plant.

**Figure 1.**
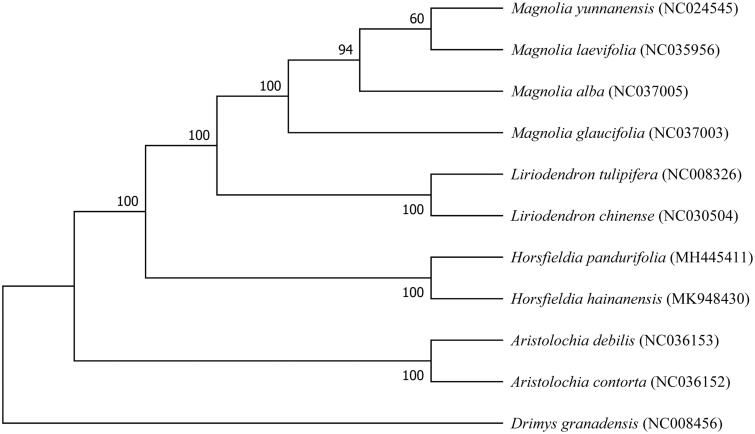
Maximum likelihood tree based on the sequences of 11 complete chloroplast genomes from order Magnoliales, with *Drimys granadensis* as outgroup.
